# SOCIOECONOMIC INEQUALITIES IN HEALTHY AGING OF OLDER KOREAN ADULTS: AN AGE-PERIOD-COHORT ANALYSIS

**DOI:** 10.1093/geroni/igae098.4080

**Published:** 2024-12-31

**Authors:** Yunhwan Lee, Eunsaem Kim, Jinhee Kim, Sukyung Kim, YooGyung Shin, Jimin Han

**Affiliations:** Ajou University School of Medicine, Suwon, Kyonggi-do, Republic of Korea; Ajou University School of Medicine, Suwon, Kyonggi-do, Republic of Korea; Ajou University School of Medicine, Suwon, Kyonggi-do, Republic of Korea; Ajou University School of Medicine, Suwon, Kyonggi-do, Republic of Korea; Ajou University School of Medicine, Suwon, Kyonggi-do, Republic of Korea; Ajou University School of Medicine, Suwon, Kyonggi-do, Republic of Korea

## Abstract

As South Korea’s healthy life expectancy continues to increase, reaching 73 years in 2021 and ranking third in the world, there is growing interest in assessing functional ability as well as its disparity across various older population segments. The study aimed to investigate healthy aging trends by socioeconomic status in the recent decade among Koreans aged 65 years or older and to analyze the age, period, and cohort effects. Data were from the four waves, about 10,000 people in each wave, of the National Survey of Older Koreans conducted every three years from 2011 to 2020. The physiological, physical, cognitive, psychological, and social dimensions were combined to create the healthy aging index (HAI), with higher scores indicating a healthier state. Socioeconomic status included education, household income, and wealth. A multilevel analysis with a cross-classified random effects model was performed. An overall trend of healthy aging in the past decade was evident, but the gap between the highest and lowest levels of education has widened. The 1935-1944 birth cohort showed a significant decrease in HAI, suggesting a long-lasting negative effect of early life adversity in the latter years of colonial rule. There was a strong age effect, demonstrating a quadratic decline in HAI over the life course. Even though the last ten years have shown a general trend toward healthy aging, socioeconomic disparity persists. The study’s findings underscore the need for policies on healthy aging to consider the life-long vulnerability of certain aging population segments characterized by adverse early life experiences.

